# Sylvatic Mosquito Diversity in Kenya—Considering Enzootic Ecology of Arboviruses in an Era of Deforestation

**DOI:** 10.3390/insects11060342

**Published:** 2020-06-03

**Authors:** Gillian Eastwood, Rosemary C. Sang, Joel Lutomiah, Philip Tunge, Scott C. Weaver

**Affiliations:** 1 Institute for Human Infections and Immunity, Center for Tropical Diseases, Department of Microbiology and Immunology, University of Texas Medical Branch, Galveston, TX 77555, USA; 2 College of Agriculture & Life Sciences, Virginia Tech, Blacksburg, VA 24060, USA; 3 Centre for Viral Research, Kenya Medical Research Institute, Mbagathi Way, Nairobi, Kenya; Rosemary.Sang@usamru-k.org (R.C.S.); joel.lutomiah@usamru-k.org (J.L.); tungephilip@gmail.com (P.T.); 4 World Reference Center for Emerging Viruses and Arboviruses, Institute for Human Infections and Immunity, Center for Tropical Diseases, Department of Microbiology and Immunology, University of Texas Medical Branch, Galveston, TX 77555, USA; sweaver@utmb.edu

**Keywords:** mosquito, disease, vector, East Africa, arbovirus, Sylvatic, species diversity, distribution, emerging

## Abstract

As new and re-emerging vector-borne diseases are occurring across the world, East Africa represents an interesting location, being the origin of several arboviruses with a history of urbanization and global spread. Rapid expansion of urban populations and alteration of natural habitats creates the opportunity for arboviruses to host-switch from wild, sylvatic hosts or vectors into urban transmission affecting human populations. Although mosquito surveillance regularly takes place in urban areas of Kenya, for example identifying vectors of dengue virus or malaria viruses, little work has been carried out to determine the distribution and abundance of sylvatic vectors. Here, we describe the mosquito vector species and diversity collected at twelve forest habitats of rural Kenya. We conducted arbovirus screening of over 14,082 mosquitoes (47 species, 11 genera) as 1520 pools, and detected seven viruses (six bunyaviruses, and one flavivirus-bunyavirus co-infection) isolated from pools of *Aedes dentatus,* *Anopheles funestus*, *Culex annulioris,* and *Cx. vansomereni*. Awareness of sylvatic vector species and their location is a critical part of understanding the ecological foci and enzootic cycling of pathogens that may be of concern to public, animal or wildlife health. As natural ecosystems come under anthropogenic pressures, such knowledge can inform us of the One Health potential for spillover or spillback leading to outbreaks, and assist in vector control strategies.

## 1. Introduction

The occurrence of vector-borne diseases has been observed in East Africa for several centuries, associated with a variety of etiological agents, host reservoirs and severity of outcome in humans or animals. For example, Crimean–Congo hemorrhagic fever caused by a tick-borne nairovirus is frequently fatal [1]; protozoa of *Plasmodium* spp., spread by mosquitoes, continue to cause malaria in certain regions [2]; East coast fever, caused by a tick-borne protozoan (*Theileria* spp.), affects livestock, and has a huge economic impact in cattle-producing regions [3]; and lymphatic filariasis, caused by a vector-borne nematode infection, has a major public health burden in multiple African countries [4]. Focusing specifically on mosquito-borne viruses, medically and veterinary important arboviruses such as Rift Valley fever virus [RVFV] are found in the region [5]. Further examples include West Nile virus [WNV], transmitted between birds and mosquitoes [6]; Yellow Fever virus [YFV] [7]; and the alphavirus o’nyong-nyong virus [ONNV], associated with sporadic but massive outbreaks of arthralgic disease recognized in East Africa since the 1950s [8,9].

For many arboviruses in East Africa, the emergent source of an epidemic is not known, but experience elsewhere shows that enzootic cycles outside the urban transmission exist. For example in Senegal, the alphavirus chikungunya [CHIKV] circulates between arboreal *Aedes* (*Stegomyia*) spp. mosquitoes and wild non-human primates [NHPs], with occasional (typically every 7–8 years) spillover into local human populations [10]. Such cycles, however, are undocumented in East Africa. Similarly, sylvatic cycles of dengue virus [DENV] have been identified in Malaysia and West Africa [11]. Despite human outbreaks caused by this flavivirus having occurred in parts of Kenya, Tanzania (Dar el Salaam), and Somalia (Mogadishu) [12,13], enzootic transmission of DENV has not been evidenced in East Africa; nevertheless, we recently detected antibodies against it in NHPs of coastal Kenya [14]. In Uganda, YFV outbreaks in 2016 were attributed to the proximity of agricultural activities to forests and swamps with sylvatic monkeys and *Aedes* spp. mosquitoes [7].

The rapid expansion of tropical urban populations and elimination of natural habitats place selective pressures on arboviruses to host-switch or adapt for human-to-human transmission [15]. This is facilitated by arboviruses predominantly being RNA viruses, which exhibit genetic plasticity and high mutation rates [16]. In addition, deforestation, land-use changes, and encroachment on protected areas occur in East Africa, as they do elsewhere in the world. Anthropogenic activities, which can impact an ecosystem in multiple ways, including a crossover of humans, domestic animal and wildlife pathogens, can lead to the emergence of infectious disease in any of these groups [17].

Kenya itself is a location of high virus discovery, for example Garissa and Ngari viruses [18], and several major vector-borne disease epidemics, e.g., Yellow Fever outbreak in 1992–1993, kala-azar (spread by sandflies) in 1977–1979 and 2014 [19,20], and is therefore a prime area to investigate enzootic sources of pathogens—knowledge of which can aid understanding of the spillover potential to human populations. Previous studies in Kenya have detected several mammalian viruses in mosquitoes including WNV, RVFV, Sindbis and Ndumu virus [21,22,23], as well as insect-specific viruses [24]. Kenya is a country in East Africa with a huge variety of habitat, spanning both coastal and lakeside (Lake Victoria), elevations from 0 to 5.197 m, and ranging from arid desert to wetlands, riverine ecosystems, tropical forest, grassland, urban communities, agriculture and rocky mountain. Although plenty of studies have looked at mosquito species in Kenya [25,26,27], these investigations have tended to focus on urban, peri-domestic or farm vectors in anthropogenic habitat, and a knowledge gap lies in addressing the vectors of forest habitat, and/or those involved in sylvatic maintenance of arboviruses.

We recently reported evidence of exposure to CHIKV in wild NHPs in Kenya [14], supporting the concept of sylvatic transmission cycles of this and other arboviruses in the region. However, characterization of the enzootic mosquito role within these cycles remains lacking, and was the initial motivation for this study of vector. CHIKV is a key example of an arbovirus emerging, spreading and modifying to adapt to new vectors, and possibly new hosts. It re-emerged in 2004 from an unknown, and potentially enzootic, source in Kenya, with a huge outbreak and high morbidity in the coastal region [28]. Traditionally transmitted in an urban cycle by *Aedes aegypti* (a peri-domestic vector species), this 2004 strain of CHIKV underwent a series of mutations in its envelope glycoprotein genes that facilitated transmission by the invasive mosquito *Ae. albopictus*, and a chain of outbreaks across the Indian Ocean Islands and Asia [29,30]. The preceding goal of this study was to detect an enzootic source of CHIKV in Kenya.

Understanding the composition of sylvatic mosquito communities is key to identifying vector diversity and pathogen maintenance in nature. We took an approach to isolate infectious virus particles to be more informative of circulating pathogens than studies that report only molecular evidence of viral RNA. In this paper, we assess the abundance and species richness of mosquitoes at forest sites, and we also sought to identify foci for enzootic transmission of arboviruses where spillover events could occur that lead to urban outbreaks.

## 2. Materials and Methods

### Mosquitoes

**Figure 1:**
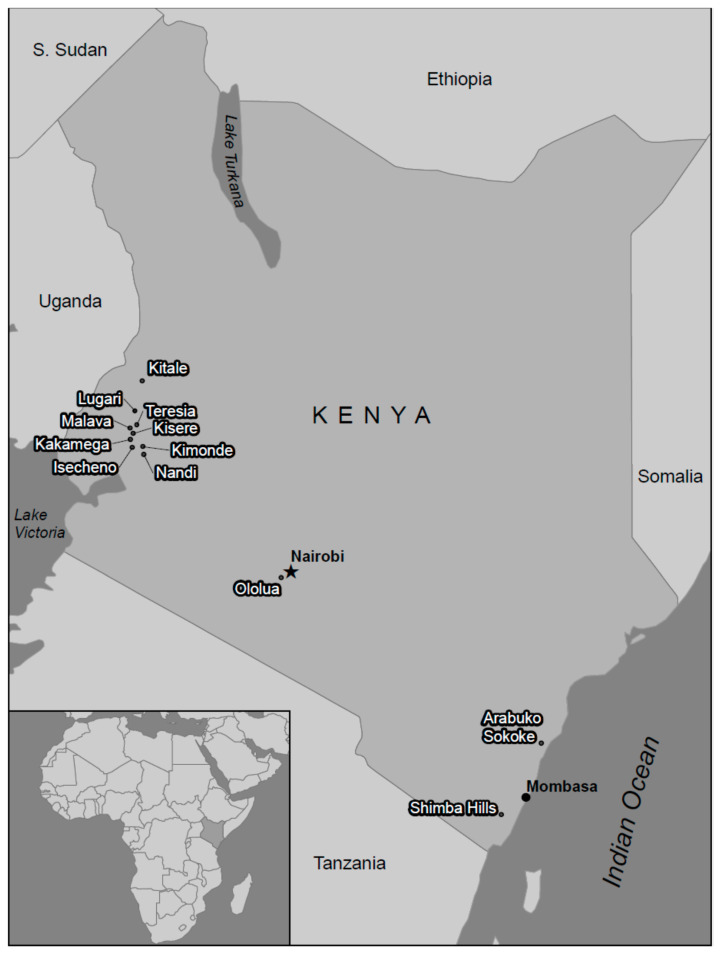
Map of Kenya, with collection sites indicated.

During 2013 and 2014, mosquitoes were collected at 12 forested or thicket sites in Western, Coastal or Central Kenya away from current human residences (Figure 1). Sites were chosen, at elevations below 2000 m (to make detection of mosquitoes more likely, since they are sensitive to cold temperatures), based on the presence of wild animals, in particularly NHPs (the suspected enzootic reservoir of CHIKV and ONNV), and a distance of over 2 km from human activity. They were also chosen according to available permits from Kenya Forestry Service, Kenya Wildlife Service, or local permissible access. Sampling effort varied at each site due to the objectives of a larger study seeking CHIKV isolates; results are therefore weighted accordingly. Mosquitoes were collected at the sites using a combination of trap types: BG Sentinel (baited with BG Lure), CDC light trap (baited with CO_2_ from a dry-ice source), and CDC gravid traps (using fermented grass or mango leaf-infused water as bait), as well as resting boxes, and hand-held aspirators.

Traps were set in the early afternoon and overnight on a transect at each site (traps were spaced over 200 m apart, usually at a height of 1.5 m, with some canopy trapping also conducted using BG or Light traps elevated on a rope pulley to approximately 20 m). Geographic coordinates for each collection point were recorded using a hand-held Global Positioning System (GPS) unit (Garmin International, Olathe, KS, USA) in WGS84 datum, and imported into ArcView GIS software (ESRI, Redlands, CA, USA). Trap contents were retrieved at dawn. Captured mosquitoes were separated from other insects, stored at −80 °C and transported to the Kenya Medical Research Institute (KEMRI) in Nairobi, where they were identified to species using a chill table and taxonomic keys [31]. Mosquitoes were pooled (up to 25 individuals per pool) according to collection date, location, and species, for subsequent viral screening.

### Virus Detection and Identification

Mosquito pools were initially examined using cell culture, since this is more efficient than going straight to molecular analysis when processing a large volume of mosquitoes for which viral prevalence rates are low. Pools were homogenized in 1 mL of Dulbecco’s Minimum Essential Medium (Gibco, Gaithersburg, MD, USA) with 10% fetal bovine serum, fungicide and antibiotics added, then centrifuged at 10,000 rpm for 4 min. The resulting supernatant was removed and 50 μL was used to inoculate sub-confluent cell monolayers in 12-well plates (Costar, Corning, NY, USA). Negative (media-only) controls were included. The two cell lines used were Vero E6 (African Green Monkey kidney) and C6/36 (*Aedes albopictus*), maintained at 37 °C and 28 °C, respectively, with 5% CO_2_. Cells were monitored for the development of cytopathic effects [CPE] for up to 12 days. Samples showing CPE were passaged twice in the same cell line for virus confirmation and amplification, prior to harvest and storage at −80 °C. Viruses were identified via RT-PCR following RNA extraction using a QIAamp Viral RNA mini kit (Qiagen, Germantown, MD, USA) according to the manufacturer’s instructions, using generic primers specific for alphaviruses, orthobunyaviruses and flaviviruses (Table 1). Viral RNA from each genus group and water were used as positive and negative controls, respectively. RNA was used as a template for cDNA synthesis with 5X first strand buffer, dNTP (10 mM), DTT (100 mM), random hexamer, RNase inhibitor (40 U/μL), and reverse transcriptase. The cycling conditions were as follows: 70 °C for 10 min, 4 °C for 5 min, 25 °C for 15 min, 42 °C for 50 min, 70 °C for 15 min, and then held at 4 °C. RT-PCR amplification was performed on 2 μL of the resulting cDNA, in a 25 μL volume of Amptaq mastermix, primers and water. Further virus identification, beyond family, was not available due to limited resources for this project.

### Statistical Analysis

Species richness (S), the total number of species detected, was calculated for each site. Species diversity and evenness of mosquito abundance was calculated using Shannon’s *H,* as used previously in [32]. Due to uneven sampling effort between sites, collections at each site were standardized using species accumulation curves based on the number of individuals caught. Validity of this method has been verified as a better weighting of effort to compare species communities than number of nights, and also avoids problems from differences in trap efficacy [33]. A modification of a randomized species accumulation curve was made using Estimate S, as per Wilcott (1999), to provide a comparative estimate of the rarefied species richness that was independent of sample size [34,35]. Arboviral infection rate (MIR) in positive mosquito species was computed for cytopathic viruses using maximum likelihood estimates (MLEs) of pooled samples (infection rate per 1000 mosquitoes) as described by Eastwood et al. (2016), via Excel© Add-In software available at http://www.cdc.gov/westnile/resourcepages/mosqSurvSoft.html [32,36].

## 3. Results

### 3.1. Mosquito Species

**Figure 2:**
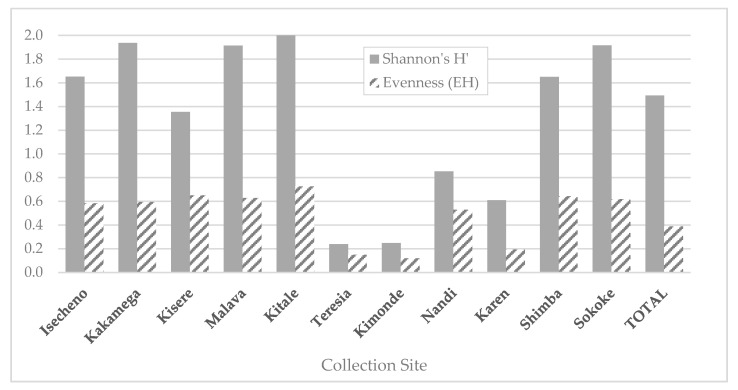
Species diversity (Shannon’s H’) and evenness (H’ divided by natural log of species richness; value 0 to 1) for mosquito species collected at ten sites in Kenya.

A total of 14,082 mosquitoes (11 genera and 47 species) were collected across 12 sylvatic sites in Kenya. Genera included *Aedeomyia*, *Aedes*, *Anopheles*, *Culex*, *Coquillettidia*, and *Mansonia*, and several of the mosquito species collected are known vectors for arboviruses or for malaria, for example *Aedes mcintoshi*, *Anopheles funestus*, *Cx. pipiens*, and *Mansonia africana*. Table S1 indicates the species and numbers of all mosquitoes captured during the study. *Culex vansomereni* (58%) represented the majority of sylvatic mosquitoes captured in total during the study, detected at all sites. Other predominant mosquito species varied according to site (Table 2). The commonest species collected at the coastal sites were *Cx. annulioris* (Arabuko-Sokoke forest) and *Cx. pipiens* (Shimba Hill Reserve). *Aedes chaussieri* was also a dominant coastal site species, and one which was absent from collections from Western Kenya. In contrast, *Coquillettidia fuscopennata* and *Cx. annulioris* were the second most prevalent species after *Cx. vansomereni* captured at sites in Western Kenya. *Aedes cumminsi* accounted for the majority of mosquitoes collected near Kitale, followed by *Cx. pipiens*. Large captures of *Cx. vansomereni* were made at Rift Valley sites, with *Cx. univittatus* as the next populous species. At the forest site near Nairobi (Ololua), *Cx. vansomereni* accounted for 88% of catches, and *Aedes tricholabis* and *Aedes dentatus* were next prevalent. The species richness and species diversity (as calculated using Shannon indices) for each site are displayed in Table 2, with evenness illustrated in Figure 2. The highest catch abundance was seen at the Nandi Forest site, near the village of Mwein, with nearly 5000 individuals captured, followed by Kimondi, also part of Nandi forest.

### 3.2. Virus Detection

Collected mosquito specimens were screened as 1520 pools on both mammalian (Vero) and mosquito (C6/36) cell lines. One flavivirus (family *Flaviviridae*) and five orthobunyaviruses (family *Bunyaviridae*) were isolated from pools representing four different species of mosquitoes, using Vero cells (Table 3). One of the positive orthobunyavirus samples came from a single male *Anopheles* spp. mosquito. A further orthobunyavirus was isolated from a *Cx. vansomereni* pool using C6/36 cells. All positive mosquito pools were captured in the central region of Kenya located near Nairobi. The flavivirus was from the same pool of *Cx. vansomereni* mosquitoes from which an orthobunyavirus was detected. The pool size was 25 individual mosquitoes and we are unable to state whether this co-infected status came from the same mosquito.

Infection prevalence (per 1000 mosquitoes) for arbovirus among the total number of that species screened were MLE = 1.1 (0.3, 3.0) (*n* = 2693 *Cx. vansomerenii*), MLE = 86.4 (5.4, 344.6) (*n* = 11 *An. funestus*), MLE = 24.2 (1.3, 122.2) (*n* = 45 *Ae. dentatus*), and MLE = N/A (single positive pool, *n* = 1 *Cx. annulioris*). Since all virus-positive pools were detected from the Central Kenya site, the above rates only considered the mosquito pools captured there, rather than from all sites, to limit heterogeneity.

## 4. Discussion

Since mosquito community composition plays a major role in the transmission of zoonotic vector-borne pathogens, it is important to consider the sylvatic mosquito species that may be encountered within forests. Changes are rapidly taking place in East African ecosystems, which leads to increased human–wildlife interaction—for example, use of agriculture space, forest resources (wood, hunting) and/or leisure (tourism), and encounters for spillover or spillback between sylvatic and urban–rural cycles of arboviral transmission are possible.

The concept of arboviruses being maintained in sylvatic cycles outside of periodic epidemic transmission affecting humans (for example, YFV maintained by *Hemagogus* spp. or *Sabethes* spp. mosquitoes in a cycle independent from urban transmission), together with an outbreak of CHIKV in Kenya from an unknown enzootic source, prompted our investigations of mosquito diversity and the prevalence of arboviruses in sylvatic habitats. The rapid modification of sylvatic habitats, or increased contact with man, reinforces the importance of such a study; Weaver (2006), for example, highlights that many zoonotic arboviruses circulate in tropical forest habitats that are being disturbed for logging and agricultural operations [15].

Recently, we provided evidence of arbovirus exposure in non-human primates in Western Kenya forests [14]. However, no isolates of CHIKV were obtained from mosquitoes of those regions during the current study, conducted at a similar time period. In fact, despite screening over 14,000 mosquitoes from 12 forest sites for evidence of either mammalian or insect-specific viruses, we found little evidence of arboviral infection in specimens from forested areas in Kenya, despite some of the mosquitoes collected being known vector species elsewhere (see below). Only one of the forest sites sampled (nearest to Nairobi) yielded virus-positive mosquitoes, with detection of six *Orthobunyavirus* isolates, and one same pool of mosquitoes also being flavivirus positive, indicating co-infection status with that pool. The *Orthobunyavirus* genus has the greatest species diversity of known virus genera, with widely distributed arthropod-borne viruses found in both hemispheres. Bunyaviruses are capable of infecting a wide range of vertebrate hosts, and include some important viral pathogens, which cause diseases of veterinary and human concern [37,38]. Elsewhere in Kenya, numerous bunyaviruses have been detected, with evidence in both mosquitoes and livestock [39,40,41]. Most *Orthobunyavirus* activity in Kenya has been detected in the Northeast region, which is dry ecosystem, with little forested habitat except alongside the Tana River. Flaviviruses elsewhere have spilled over from their enzootic cycle—for example, WNV has frequently left its bird–mosquito transmission cycle to infect horses and humans (dead-end hosts) via a bridge-vector mosquito, resulting in neurological infections [42]. Regarding the likelihood of false negatives during screening, it is possible that low-level viral RNA exists within mosquitoes. However, the passaging of samples within cell culture was expected to amplify infectious viral particles to a detectable level should they exist.

Species richness of the sylvatic mosquito community in the Kenya region sampled is high; 47 different species were collected, illustrating the high diversity of sylvatic mosquitoes. Sites in Rift Valley were less diverse, and showed unequal evenness, i.e., that key species dominated, rather than an equal spread of species.

To discuss some of the more notable vector species within sylvatic sites of Kenya, we highlight (i) *Aedes* (*stegomyia*) spp., e.g., *Aedes africanus* and *Ae. vittatus*, which are arboreal mosquitoes found to be competent for CHIKV transmission [43], and associated with a number of other alpha- and flavi-viruses [44], and *Ae. aegypti* as a key vector of CHIKV, YFV, DENV (although the latter was only detected at one forest site). (ii) *Aedes cumminsi* is believed to be a vector of Spondweni virus and RVFV [45], and an isolate of Middleburg virus was made from a pool of *Ae. cumminsi* from forests in Senegal (Robin, 1969). (iii) *Culex* spp. are often associated with WNV transmission and represented a large proportion of the mosquitoes collected, in particular *Cx. vansomereni* (>58% of catches in our study). This species was found to be a competent vector of WNV in the laboratory [46]. (iv) In neighboring Uganda, *Ae. tarsalis* and *Coq. fuscopennata* (both found in the study here) were sources of Pongola virus, which can cause febrile illness in humans [47]. (v) *Anopheles* spp. are generally associated with transmission of malaria. However, *An. funestus* and *An. gambiae* are also vectors of ONNV, and this genera of mosquito is increasingly detected with arboviruses; transmission capability requires determination [9]. That a bunyavirus was detected here in a male *An. funestus,* suggests that vertical transmission is occurring as a mechanism for virus persistence.

Mosquito studies of sylvatic habitats with potential for spillover under habitat disturbance elsewhere show a similar incidence of both disease vectors and lesser known species. In Senegal, tree-hole breeding species such as *Ae. luteocephalus*, *Ae. furcifer*, and *Ae. taylori* (all associated with *Aedes*-borne viruses) predominated at forest sites [48]. Mosquito community studies elsewhere have been used to suggest whether outbreaks of prominent arboviruses could be supported [49,50]. Knowing more about vectorial capacity, including vector competence, of sylvatic species in Kenya would suggest whether the elements exist to support enzootic outbreaks. Other arboviruses, although with little known about behavior or potential risk, have been detected from mosquito species captured during our study—for example, Kamese virus (family *Rhabdoviridae*) was first detected from *Ae. annulioris* in forests in Uganda [45,51]. Mosquito diversity in forests of West Africa has been demonstrated to include many *Aedes* spp., including several detected with medically important arboviruses including CHIKV [10]. Although no vector evidence of CHIKV was detected here, we previously showed the virus to be circulating in the region via vertebrate–host studies [14]. Further mosquito studies, incorporating improved methodology to detect *Aedes stegomyia* spp., are recommended.

Between-site habitat differences, weather, and longer seasonal fluctuations can influence results. Although efforts were made to sample throughout the year, rainfall is higher during March–June and October–November. Several mosquitoes, especially those whose morphological quality deteriorated in large and wet overnight catches, could only be identified to genus level here, and so, although *Cx. vansomereni* was suspected for many *Culex* spp., further distinction of diversity was prevented. The highest nightly catch abundances were made at sites in Western Kenya, which were mainly sampled during the month of March 2014. However, other sites sampled in that same month (including Ololua) did not reflect such high abundance. Nevertheless, seasonality is an important factor that should be acknowledged in the design of future studies, where the main objective is comparative abundance of these mosquito species. In Senegal, mosquito diversity was higher during months when more water in larval habitat was likely [48]. Here, we combined a variety of sampling opportunities (e.g., alongside other wildlife studies) and therefore exact comparison of seasonal effects upon vector species was not possible.

We focus on forested habitat, but ecosystems still vary considerably. Unequal sampling was accounted for, however some sites noticeably yielded smaller sample sizes, e.g., few mosquitoes were obtained from Shimba Hills Reserve (Kwale County), reflecting the hot dry ecosystem, with fewer areas of forest (a more savannah ecosystem), plus samples were taken from there in the dry season (as an aside to a primate study). In the coastal Malindi region of Kenya, Mwangangi et al. (2012) found *Cx. quinquefasciatus* to be the vastly predominant species, especially in urban and peri-urban strata. There, most vectors—*An. gambiae*, *An. funestus* and *An. coustani*—were found in the ‘rural’ stratum, which was defined, however, as agricultural land rather than intact forest [25]. There are challenges comparing species inventories of sites where there is no indication of how complete the inventory is, or when different methods have been used.

In Kenya, it is now difficult to locate primary (unaltered) tropical forest; Kakamega forest being the only existent remnant of the Guinea–Congolion rainforest belt that spanned equatorial Africa [52], and even then Kakamega forest is frequented by local villagers and popular for ecotourism. Braack et al. (2018) highlight that increased land transformation in Africa and disruption of historical ecological processes promote contact between humans and infected wildlife or sylvatic vectors [45]. A framework presented by Faust et al. (2018) suggests that the highest spillover risk occurs at intermediate levels of habitat loss, whereas the largest, but rare, epidemics occur at extremes of land conversion [53]. Tropical forest habitat is generally reported as threatened due to encroachment for new land for agriculture and settlement and overharvesting of forest resources. In other countries, although undisturbed primary forest has peak mosquito diversity, disturbance has led to mosquito communities with a greater abundance of ‘pathogen-transmitting’ vector species [54]. This highlights that it is the specific mosquito species that is present that matters, not the quantity or richness in general. Elsewhere, clearing patches of tropical forest often appears to create ideal habitat along forest edges for malaria mosquito vectors to breed, and deforestation is associated with increases in disease through ecological mechanisms [55,56]. Indeed, Afrane et al. (2012) highlight how deforestation in the western Kenyan highlands is associated with increased development and survival of some *Anopheles* vectors [57].

Host-switching of pathogens, from one vector species to another, or one host species to another, is a common event during long-term arbovirus evolution [15,58], and one we should attempt to anticipate. Taxonomic identification of sylvatic mosquitoes can be a challenging process since many entomological specialists are not as familiar with such species; however, continued monitoring of mosquito vectors is recommended, and not just around towns and villages where surveillance and urban transmission most commonly takes place. For example, supplementation of activities conducted by KEMRI or Ministries of Health to include regions where rural workers or tourists may come into contact with sylvatic cycles of disease pathogens may be useful to avert emergent outbreaks occurring via spillover.

Most arboviruses are zoonotic, and understanding sylvatic transmission of vector-borne pathogens is not complete without determination of the host reservoir involved in their enzootic maintenance. Studies of sylvatic vector species should be complemented by understanding of enzootic vertebrate host interaction—for example, by mosquito blood-meal analysis, viral or serological surveys.

## 5. Conclusions

In summary, a wide diversity of mosquito species exists outside of urban or peridomestic settlements in East Africa—many of which are known as competent disease vectors elsewhere. As humans encroach on forests and previously undisturbed sylvatic habitats, awareness of arthropod vector species and their pathogen transmission role is vital to limit the potential for disease outbreak. We demonstrate the high species diversity of mosquitoes across sylvatic Kenyan ecosystems, and encourage that their role in pathogen maintenance networks is investigated further. Recognizing the existence of sylvatic mechanisms of arbovirus proliferation is an essential component to One Health, with forest degradation, spillover and spillbacks between domesticated and wild species (arbitrated by non-urban arthropod vectors) relevant for both public, animal and wildlife health.

## Figures and Tables

**Table 1:** Molecular primers and cycling conditions used to test samples.

Virus	Primer	Primer Sequence	Cycling Conditions			
Alphavirus	Vir2052F	TGG CGC TAT GAT GAA ATC TGG AAT GTT	95 °C (10 min)	95 °C (30 s) 49 °C (30 s) 72 °C (30 s) for 35 cycles	72 °C (10 min)	4 °C (hold)
	Vir2052R	TAC GAT GTT GTC GTC GCC GAT GAA				
Flavivirus	Fu1	TAC AAC ATG ATG GGA AAG AGA GAG AA	95 °C (10 min)	95 °C (30 s) 55 °C (30 s) 68 °C (45 s) for 35 cycles	72 °C (7 min)	4 °C (hold)
	CDF2	GTG TCC CAG CCG GCG GTG TCA TCA GC				
Ortho-bunyavirus	BCS82c	ATG ACT GAG TTG GAG TTT CAT GAT GTC GC				
	BCS332v	TGT TCC TGT TGC CAG GAA AAT				

**Table 2:** Mosquito capture, species richness and species diversity by sample site.

Region	Site	Effort (Nights)	Month Sampled	No. Individual Mosquitoes Collected	Species Richness	Predominant Species at the Site			Species Diversity
Western	**Isecheno**	7	August	427	17	*Culex vansomereni*	*Coquillettidia fuscopennata*	*Culex annulioris*	1.652
	**Kakamega**	6	Aug/Sept	1363	26	*Culex vansomereni*	*Coquillettidia fuscopennata*	*Culex pipiens*	1.936
	**Kisere**	1	March	112	8	*Culex vansomereni*	*Coquillettidia fuscopennata*	*Culex pipiens*	1.354
	**Malava**	2	March	454	21	*Culex vansomereni*	*Aedes aegypti*	*Culex annulioris*	1.914
	**Kitale**	2	March	340	16	*Aedes cumminsi*	*Culex pipiens*	*Culex vansomereni*	2.011
Rift Valley	**Teresia**	1	March	375	5	*Culex vansomereni*	*Culex annulioris*	*Anopheles funestus*	0.239
	**Kimonde**	2	March	2203	8	*Culex vansomereni*	*Culex univittatus*	*Culex bitaeniorhynchus*	0.248
	**Lugari**	1	March	20	3	*Culex vansomereni*	*Aedes cumminsi*	*-*	-
	**Nandi**	1	March	4927	5	*Culex vansomereni*	*Culex univittatus*	*Culex pipiens*	0.852
Central	**Ololua**	9	Feb/March	3066	23	*Culex vansomereni*	*Aedes tricholabis*	*Aedes dentatus*	0.609
Coastal	**Shimba**	4	December	98	13	*Culex pipiens*	*Culex vansomereni*	*Culex zombaensis*	1.650
	**Sokoke**	10	July/August	697	22	*Culex annulioris*	*Culex vansomereni*	*Aedes chaussieri*	1.915

**Table 3:** Mosquito species pools that yielded viral isolates.

Virus Type	Mosquito Species	Pool Size (No. of Mosquitoes)	Cell Line
Orthobunyavirus	*Culex annulioris*	1	Vero
	*Culex vansomereni*	1	Vero
	*Anopheles funestus*	1 [male]	Vero
	*Aedes dentatus*	12	Vero
	*Culex vansomereni*	25	C6/36
Flavivirus and Orthobunyavirus co-infection	*Culex vansomereni*	25	Vero

## Supplementary Materials

The following are available online at https://www.mdpi.com/2075-4450/11/6/342/s1: Table S1: Mosquito species collected at sylvatic sites in Kenya.

## References

[1] Lwande O.W., Irura Z., Tigoi C., Chepkorir E., Orindi B.O., Musila L., Venter M., Fischer A., Sang R. (2012). Seroprevalence of Crimean Congo hemorrhagic fever virus in Ijara District, Kenya. Vector Borne Zoonotic Dis..

[2] Conn J.E., Norris U.E., Donnelly M.J., Beebe N.W., Burkot T.R., Coulibaly M.B., Chery L., Eapen A., Keven J.B., Kilama M. (2015). Entomological monitoring and evaluation: Diverse transmission settings of ICEMR Projects will require local and regional malaria elimination strategies. Am. J. Trop. Med. Hyg..

[3] Gachohi J., Skilton R.A., Hansen F., Ngumi P., Kitala P.M. (2012). Epidemiology of East Coast fever (Theileria parva infection) in Kenya: Past, present and the future. Parasites Vectors.

[4] Mwandawiro C.S., Fujimaki Y., Mitsui Y., Katsivo M. (1997). Mosquito vectors of bancroftian filariasis in Kwale District, Kenya. East. Afr. Med. J..

[5] Himeidan Y.E.S., Kweka E.J., Mahgoub M.M., El Rayah E.A., Ouma J.O. (2014). Recent outbreaks of Rift Valley Fever in East Africa and the Middle East. Front. Public Health.

[6] Nyamwaya D.K., Wang’Ondu V., Amimo J.O., Michuki G., Ogugo M., Ontiri E., Sang R., Lindahl J.F., Grace D., Bett B. (2016). Detection of West Nile virus in wild birds in Tana River and Garissa Counties, Kenya. BMC Infect. Dis..

[7] Kwagonza L., Masiira B., Kyobe-Bosa H., Kadobera D., Atuheire E.B., Lubwama B., Kagirita A., Katushabe E., Kayiwa J., Lutwama J.J. (2018). Outbreak of yellow fever in central and southwestern Uganda, February 2016. BMC Infect. Dis..

[8] LaBeaud A.D., Banda T., Brichard J., Muchiri E.M., Mungai P.L., Mutuku F.M., Borland E., Gildengorin G., Pfeil S., Teng C.Y. (2015). High rates of o’nyong nyong and chikungunya virus transmission in Coastal Kenya. PLoS Negl. Trop. Dis..

[9] Powers A.M., Brault A.C., Tesh R.B., Weaver S.C. (2000). Re-emergence of chikungunya and o’nyong-nyong viruses: Evidence for distinct geographical lineages and distant evolutionary relationships. J. Gen. Virol..

[10] Diallo M., Traore-Lamizana M., Fontenille D., Thonnon J. (1999). Vectors of Chikungunya virus in Senegal: Current data and transmission cycles. Am. J. Trop. Med. Hyg..

[11] Vasilakis N., Cardosa J., Hanley K.A., Holmes E.C., Weaver S.C. (2011). Fever from the forest: Prospects for the continued emergence of sylvatic dengue virus and its impact on public health. Nat. Rev. Genet..

[12] Kanesa-Thasan N., Chang G.J., Smoak B.L., Magill A., Burrous M.J., Hoke C.H. (1998). Molecular and epidemiologic analysis of dengue virus isolates from Somalia. Emerg. Infect. Dis..

[13] Mboera L.E.G., Mweya C., Rumisha S.F., Tungu P.K., Stanley G., Makange M.R., Misinzo G., De Nardo P., Vairo F., Oriyo N.M. (2016). The risk of dengue virus transmission in Dar es Salaam, Tanzania during an epidemic period of 2014. PLoS Negl. Trop. Dis..

[14] Eastwood G., Taracha E.L.N., Weaver S.C., Sang R.C., Guerbois M. (2017). Enzootic circulation of chikungunya Virus in East Africa: Serological evidence in non-human Kenyan primates. Am. J. Trop. Med. Hyg..

[15] Weaver S.C. (2006). Evolutionary influences in arboviral disease. Mol. Asp. Myeloid Stem Cell Dev..

[16] Holland J., Domingo E. (1998). Origin and evolution of viruses. Virus Genes.

[17] Daszak P. (2000). Emerging Infectious Diseases of Wildlife—Threats to biodiversity and human health. Science.

[18] Gerrard S.R., Li L., Barrett A.D., Nichol S.T. (2004). Ngari virus is a Bunyamwera Virus reassortant that can be associated with large outbreaks of Hemorrhagic Fever in Africa. J. Virol..

[19] Wijers D.J., Kiilu G. (1984). Studies on the vector of kala-azar in Kenya, VIII. The outbreak in Machakos District; epidemiological features and a possible way of control. Ann. Trop. Med. Parasitol..

[20] Reiter P., Cordellier R., Ouma J.O., Cropp C.B., Savage H.M., Sanders E.J., Marfin A.A., Tukei P.M., Agata N.N., Gitau L.G. (1998). First recorded outbreak of yellow fever in Kenya, 1992–1993. II. Entomologic investigations. Am. J. Trop. Med. Hyg..

[21] Ochieng C., Lutomiah J., Makio A., Koka H., Chepkorir E., Yalwala S., Mutisya J., Musila L., Khamadi S., Richardson J. (2013). Mosquito-borne arbovirus surveillance at selected sites in diverse ecological zones of Kenya; 2007–2012. Virol. J..

[22] LaBeaud A.D., Sutherland L.J., Muiruri S., Muchiri E.M., Gray L.R., Zimmerman P.A., Hise A., King C.H. (2011). Arbovirus prevalence in mosquitoes, Kenya. Emerg. Infect. Dis..

[23] Lutomiah J., Bast J., Clark J., Richardson J., Yalwala S., Oullo D., Mutisya J., Mulwa F., Musila L., Khamadi S. (2013). Abundance, diversity, and distribution of mosquito vectors in selected ecological regions of Kenya: Public health implications. J. Vector Ecol..

[24] Iwashita H., Higa Y., Futami K., Lutiali P.A., Njenga S.M., Nabeshima T., Minakawa N. (2018). Mosquito arbovirus survey in selected areas of Kenya: Detection of insect-specific virus. Trop. Med. Health.

[25] Mwangangi J.M., Midega J., Kahindi S., Njoroge L., Nzovu J., Githure J., Mbogo C.M., Beier J.C. (2011). Mosquito species abundance and diversity in Malindi, Kenya and their potential implication in pathogen transmission. Parasitol. Res..

[26] Agha S.B., Tchouassi D.P., Bastos A.D.S., Sang R. (2017). Dengue and yellow fever virus vectors: Seasonal abundance, diversity and resting preferences in three Kenyan cities. Parasites Vectors.

[27] Ajamma Y., Villinger J., Omondi D., Salifu D., Onchuru T.O., Njoroge L., Muigai A.W.T., Masiga D.K. (2016). Composition and genetic diversity of mosquitoes (Diptera: Culicidae) on islands and mainland shores of Kenya’s lakes Victoria and Baringo. J. Med. Èntomol..

[28] Sergon K., Onyango C., Breiman R.F., Ofula V., Bedno S., Konongoi L.S., Burke H., Konde J., Sang R., Dumilla A.M. (2008). Seroprevalence of chikungunya virus (CHIKV) infection on Lamu Island, Kenya, October 2004. Am. J. Trop. Med. Hyg..

[29] Tsetsarkin K.A., VanLandingham D.L., McGee C.E., Higgs S. (2007). A single mutation in chikungunya virus affects vector specificity and epidemic potential. PLoS Pathog..

[30] Tsetsarkin K.A., Chen R., Yun R., Rossi S.L., Plante K.S., Guerbois M., Forrester N., Perng G.C., Sreekumar E., Leal G. (2014). Multi-peaked adaptive landscape for chikungunya virus evolution predicts continued fitness optimization in *Aedes albopictus* mosquitoes. Nat. Commun..

[31] Edwards F. (1941). Mosquitoes of the Ethiopian Region.

[32] Eastwood G., Sanjur O.I., Pecor J.E., Loaiza J.R., Pongsiri M.J., Auguste A.J., Kramer L.D. (2016). Enzootic arbovirus surveillance in forest habitat and phylogenetic characterization of novel isolates of Gamboa virus in Panama. Am. J. Trop. Med. Hyg..

[33] Willott S. (2001). Species accumulation curves and the measure of sampling effort. J. Appl. Ecol..

[34] Willott S.J. (1999). The effects of selective logging on the distribution of moths in a Bornean rainforest. Philos. Trans. R. Soc. B Boil. Sci..

[35] Colwell R.K., Elsensohn J.E. (2014). EstimateS turns 20: Statistical estimation of species richness and shared species from samples, with non-parametric extrapolation. Ecography.

[36] Biggerstaff B.J. (2009). PooledInfRate, Version 4.0: A Microsoft^®^ Office Excel© Add-In to Compute Prevalence Estimates from Pooled Samples.

[37] Contigiani M.S., Diaz L.A., Tauro L.B., Marcondes C.B. (2017). Bunyaviruses. Arthropod Borne Diseases.

[38] Shope R. (1996). Bunyaviruses. Medical Microbiology.

[39] Odhiambo C., Venter M., Lwande O., Swanepoel R., Sang R. (2015). Phylogenetic analysis of Bunyamwera and Ngari viruses (family *Bunyaviridae*, genus *Orthobunyavirus*) isolated in Kenya. Epidemiol. Infect..

[40] Davies F.G., Jessett D.M. (1985). A study of the host range and distribution of antibody to Akabane virus (genus *bunyavirus*, family *Bunyaviridae*) in Kenya. J. Hyg..

[41] Crabtree M., Sang R., Lutomiah J., Richardson J., Miller B. (2009). Arbovirus surveillance of mosquitoes collected at sites of active Rift Valley fever virus transmission: Kenya, 2006–2007. J. Med. Èntomol..

[42] Chancey C., Grinev A., Volkova E., Rios M. (2015). The global ecology and epidemiology of West Nile virus. BioMed Res. Int..

[43] Diagne C.T., Weaver S.C., Dia I., Knight R., Guerbois M., Diallo D., Faye O., Sall A.A., Diallo M., Faye O. (2014). Vector Competence of *Aedes aegypti* and *Aedes vittatus* (Diptera: Culicidae) from Senegal and Cape Verde Archipelago for West African Lineages of Chikungunya Virus. Am. J. Trop. Med. Hyg..

[44] Díez-Fernández A., La Puente J.M., Ruíz S., Gutiérrez-López R., Soriguer R.C., Figuerola J. (2018). *Aedes vittatus* in Spain: Current distribution, barcoding characterization and potential role as a vector of human diseases. Parasites Vectors.

[45] Braack L., Almeida A., Cornel A.J., Swanepoel R., De Jager C. (2018). Mosquito-borne arboviruses of African origin: Review of key viruses and vectors. Parasites Vectors.

[46] Lutomiah J.L., Koka H., Mutisya J., Yalwala S., Muthoni M., Makio A., Limbaso S., Musila L., Clark J.W., Turell M.J. (2011). Ability of selected Kenyan mosquito (Diptera: Culicidae) species to transmit West Nile virus under laboratory conditions. J. Med. Èntomol..

[47] Mossel E.C., Crabtree M.B., Mutebi J.-P., Lutwama J.J., Borland E., Powers A.M., Miller B.R. (2017). Arboviruses isolated from mosquitoes collected in Uganda, 2008–2012. J. Med. Èntomol..

[48] Diallo D., Diagne C.T., Buenemann M., Ba Y., Dia I., Faye O., A Sall A., Faye O., Watts D.M., Weaver S.C. (2018). Biodiversity pattern of mosquitoes in southeastern Senegal, epidemiological implication in arbovirus and malaria transmission. J. Med. Èntomol..

[49] Torres R., Samudio R., Carrera J.P., Young J., Marquez R., Hurtado L., Weaver S., Chaves L.F., Tesh R., Carrera L.C. (2017). Enzootic mosquito vector species at equine encephalitis transmission foci in the República de Panamá. PLoS ONE.

[50] McMillan J.R., Armstrong P.M., Andreadis T.G. (2020). Patterns of mosquito and arbovirus community composition and ecological indexes of arboviral risk in the northeast United States. PLoS Negl. Trop. Dis..

[51] Tchetgna H.D.S., Nakoune E., Selekon B., Gessain A., Manuguerra J.-C., Kazanji M., Berthet N. (2017). Molecular characterization of the Kamese virus, an unassigned Rhabdovirus, isolated from *Culex pruina* in the Central African Republic. Vector Borne Zoonotic Dis..

[52] Wagner P., Köhler J., Schmitz A., Böhme W. (2008). The biogeographical assignment of a west Kenyan rain forest remnant: Further evidence from analysis of its reptile fauna. J. Biogeogr..

[53] Faust C.L., McCallum H., Bloomfield L.S.P., Gottdenker N.L., Gillespie T.R., Torney C.J., Dobson A.P., Plowright R.K. (2018). Pathogen spillover during land conversion. Ecol. Lett..

[54] Loaiza J.R., Dutari L.C., Rovira J.R., Sanjur O.I., LaPorta G., Pecor J., Foley D.H., Eastwood G., Kramer L.D., Radtke M. (2017). Disturbance and mosquito diversity in the lowland tropical rainforest of central Panama. Sci. Rep..

[55] Vittor A.Y., Pan W., Gilman R.H., Tielsch J., Glass G., Shields T., Sánchez-Lozano W., Pinedo V.V., Salas-Cobos E., Flores S. (2009). Linking deforestation to malaria in the Amazon: Characterization of the breeding habitat of the principal malaria vector, *Anopheles darlingi*. Am. J. Trop. Med. Hyg..

[56] Macdonald A.J., Mordecai E.A. (2019). Amazon deforestation drives malaria transmission, and malaria burden reduces forest clearing. Proc. Natl. Acad. Sci. USA.

[57] Afrane Y.A., Githeko A.K., Yan G. (2012). The ecology of Anopheles mosquitoes under climate change: Case studies from the effects of deforestation in East African highlands. Ann. N. Y. Acad. Sci..

[58] Kovalev S., Mukhacheva T.A. (2014). Tick-borne encephalitis virus subtypes emerged through rapid vector switches rather than gradual evolution. Ecol. Evol..

